# The Cracking Behavior of Two Dental Composite Materials Validated through Multifractal Analyzes

**DOI:** 10.3390/ijms24076493

**Published:** 2023-03-30

**Authors:** Irina Nica, Florin Nedeff, Valentin Nedeff, Cristina Popa, Ștefan Lucian Toma, Maricel Agop, Decebal Vasincu

**Affiliations:** 1 Department of Odontology-Periodontology, Fixed Prosthesis, Faculty of Dental Medicine, Grigore T. Popa University of Medicine and Pharmacy, 700115 Iasi, Romania; 2 Department of Environmental Engineering and Mechanical Engineering, Faculty of Engineering, Vasile Alecsandri University of Bacău, 600115 Bacău, Romania; 3 Department of Industrial Systems Engineering and Management, Faculty of Engineering, Vasile Alecsandri University of Bacău, 600115 Bacău, Romania; 4 Department of Oral Pathology, Faculty of Dental Medicine, Grigore T. Popa University of Medicine and Pharmacy, 700115 Iasi, Romania; 5 Department of Materials Engineering and Industrial Security, Faculty of Materials Science and Engineering, Gheorghe Asachi Technical University of Iasi, 700050 Iasi, Romania; 6 Department of Physics, Faculty of Machine Manufacturing and Industrial Management, Gheorghe Asachi Technical University of Iasi, 700050 Iasi, Romania; 7 Academy of Romanian Scientists, 050094 Bucharest, Romania; 8 Department of Biophysics, Faculty of Dental Medicine, Grigore T. Popa University of Medicine and Pharmacy, 700115 Iasi, Romania

**Keywords:** dental composite material, compression, non-linear behaviors, multifractality

## Abstract

The aim of this in vitro study was to analyze, both experimentally and theoretically, the mechanical behavior of two types of composite materials used in restoring dental integrity. The samples of each composite resin, namely Filtek Supreme XT (3M ESPE, St. Paul, MN, USA) and Filtek Z250 (3M ESPE, St. Paul, MN, USA), were experimentally analyzed by determining their compressive strength and fracture behavior. The fractured fragments of the samples were subjected to surface evaluation by scanning electron microscopy. The compressive stress—compressive strain dependencies revealed stronger cracking of the Filtek Supreme XT composite than Filtek Z250 prior to fracture. Theoretically, the evaluation was made by means of holographic implementations of such types of composite materials. A Hooke-type equation in a differential form is presented, which links the proposed theoretical model with the experimentally obtained data.

## 1. Introduction

The choice of materials for direct restorations is challenging, as they are subject to masticatory forces, sometimes harmful, that can lead to cracks or fractures in final restorations. In this context, mechanical properties play a central role in assessing the longevity of restorations. The diverse types of stresses during masticatory processes make it necessary to determine several mechanical characteristics of restorative composites: bending strength, radial and longitudinal compressive strength, tensile strength, etc. Among these, compressive strength is essential for the durability of restorations [1,2].

On the other hand, the usual physical models used in describing the dynamics of dental composite materials are based on the hypothesis of the differentiability of the physical quantities used to describe their evolution. Therefore, the validity of these models must be understood gradually in areas where differentiability and integrability are still functional [3,4,5]. However, when discussing nonlinearity and chaoticity in the dynamics of dental composite materials, differentiable and integrable mathematical procedures are of little use. Thus, to correctly show dental composite materials dynamics, we must add scale resolution for physical variables and fundamental equations controlling the dynamics [3,4,5].

Accepting the above affirmation, any physical variable (used in the description of dental composite materials dynamics) will depend on the usual mathematical procedures on spatial and time coordinates and on a scale resolution. Specifically, instead of working with a single physical variable (a strictly non-differentiable mathematical function), it is possible to operate only with approximations of this mathematical function, resulting in averaging it at different scale resolutions. Thus, any physical variable used to describe the dynamics of dental composite materials will operate as the limit of a family of mathematical functions, the function being non-differentiable for zero scale resolution and differentiable for non-zero scale resolution [6,7,8].

This way of describing the dynamics of dental composite materials obviously implies the development of both new geometric structures and physical theories consistent with these geometric structures, for which the laws of motion, invariant to time coordinate transformations, are also invariant to transformations with respect to scale resolution. Such a geometric structure is based on the concept of the fractal/multifractal and the corresponding physical model described in the Scale Relativity Theory (SRT) [6,7,8]. From this perspective, the holographic implementation in the description of the dynamics of dental composite materials will be explicitly made based on the description of the dynamics of the structural units of any dental composite materials by continuous but non-differentiable curves (fractal/multifractal curves). 

The present study aimed to analyze, both experimentally and theoretically, the mechanical behavior of two types of composite materials used for direct restorations: experimentally, by determining their compressive strength and fracture behavior to make a more accurate choice of composite in relation to the position of the tooth on the arch and the degree of overloading of the fillings and theoretically, using holographic implementations of such types of composite materials.

## 2. Results

**Figure 1:**
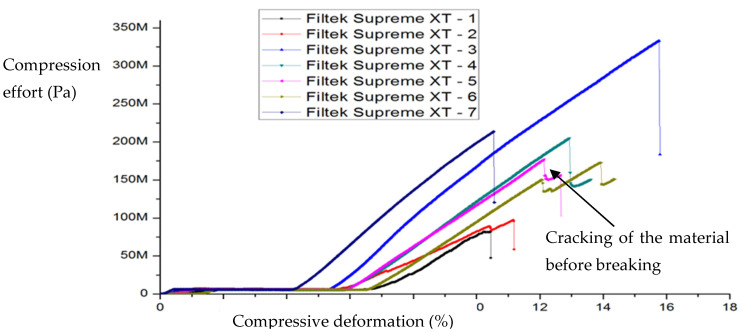
Variation of compressive strain with compressive stress for Filtek Supreme XT composite specimens.

**Figure 2:**
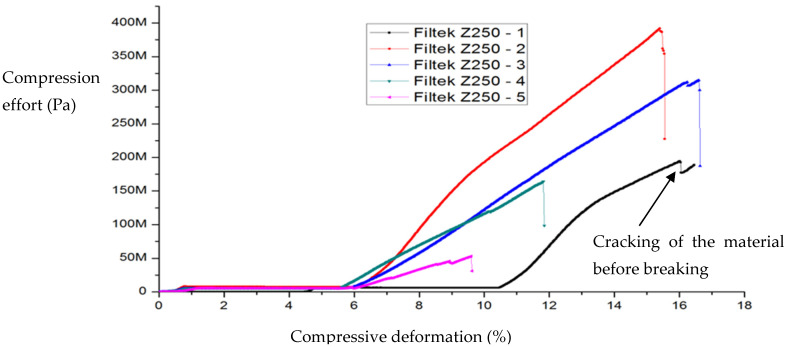
Variation of compressive strain with compressive stress for Filtek Z250 composite specimens.

The compressive stress (in MPa) was determined by relating the applied force (N) to the surface area of the base of the specimen analyzed and the relative deformation by relating its length to its initial value. Shown below are the variations of compressive strain (%) with compressive stress (MPa) for a series of specimens, Filtek Supreme XT (Figure 1) and Filtek Z250 (Figure 2), which were subjected to compression at a feed rate of 1 mm/min. The specimens did not initially show surface cracks.

All the samples showed high compressive strength; the experimental values obtained are shown in Table 1, reaching maximum forces in the order of thousands of newtons.

## 3. Discussion

### 3.1. Analysis of the Experimental Data

Table 1 shows that there are widely dispersed values for both types of material. This is because the existence of internal defects in the materials can lead to the formation of micro voids that weaken the compressive strength. Thus, it was found that very good pressing prior to light curing in the fluid state leads to compact specimens with high strength. In this context, we will note only the maximum values obtained by us, 333 MPa for Filtek Supreme XT and the higher 392 MPa for Filtek Z250. We also note that at the test speeds used, 0.5, 1, and 1.5 mm/min, no significantly different results were obtained.

Comparing with the data indicated by the manufacturer, (360±5) MPa for Filtek Supreme XT, respectively (410±20) MPa for Filtek Z250, it can be seen that our maximum data falls within these ranges.

Although the data given in the literature for compressive strength vary within very wide limits, it is unanimously accepted that all mechanical properties (compressive strength, diametral strength, flexural strength) are better for Filtek Z250 hybrid composite than for Filtek Supreme XT nanocomposite [9,10,11,12]. Both materials have the same polymer matrix and similar particle loadings but different particle sizes. Therefore, a possible explanation for these properties lies in the different propagation mechanisms of microcracks during deformation. Thus, it can be seen that the micrometer particles of the Filtek Z250 block crack propagation and reflection, while the nanometer particles allow it. In fact, the curves in Figure 1 and Figure 2, through the regions marked with dotted lines, indicate stronger cracking of Filtek Supreme XT composite than Filtek Z250 composite before breakage.

In order to highlight this tendency of microcrack blocking on micrometric particles, the surface of the material in fracture was studied by SEM microscopy.

**Figure 3:**
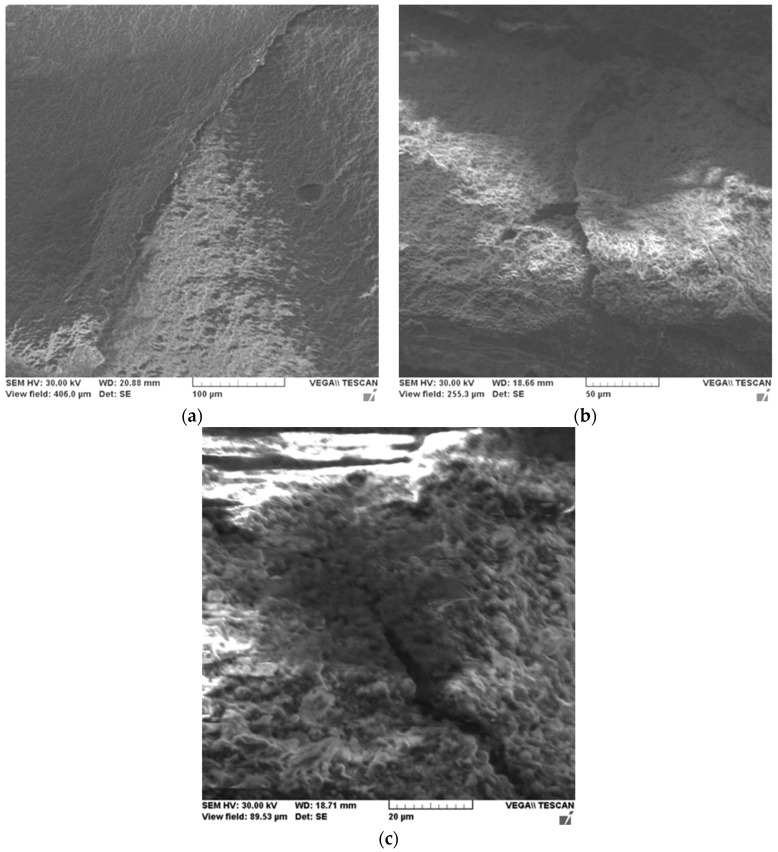
SEM microscopies of the Filtek Supreme XT-2 sample at various magnification powers: 700× (**a**), 1100× (**b**) and 3200× (**c**).

Figure 3 shows microstructural details of the Filtek Supreme XT-2 sample at various magnification powers.

Fracture surface analysis of Filtek Supreme XT material under compressive stress shows brittle fracture of the material with the propagation of microcracks along the fracture planes. It is observed from the micrographs the direction of material pull-out is characterized by overlapping planes of the material, major cracks oriented along the same direction, and areas with effective micro filler pull-outs.

**Figure 4:**
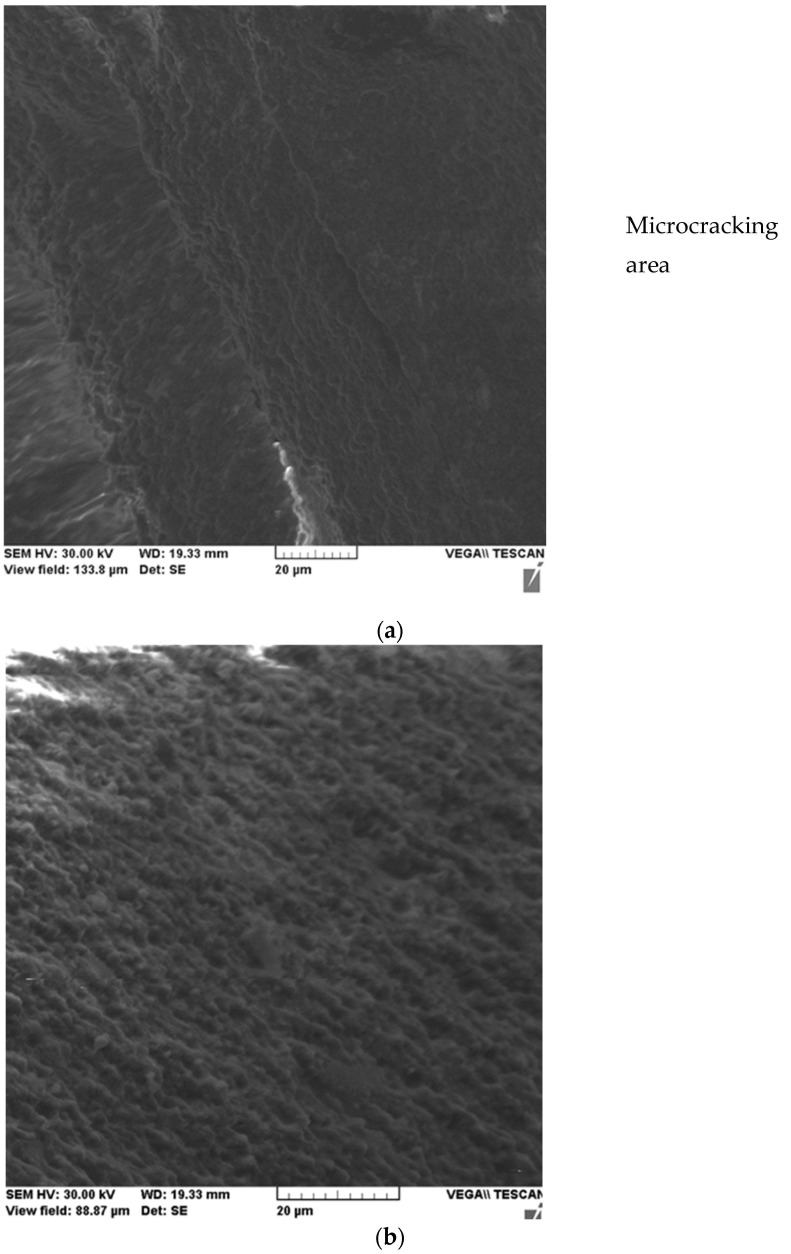
SEM microscopy of the Filtek Supreme XT-4 sample with propagation of a microcrack between nanometer particle clusters (**a**) and an unaffected area (**b**).

Detailing a microcrack, Figure 4a shows its linear propagation through micro clusters of nanometer particles and adjacent areas of unaffected material are shown in Figure 4b.

**Figure 5:**
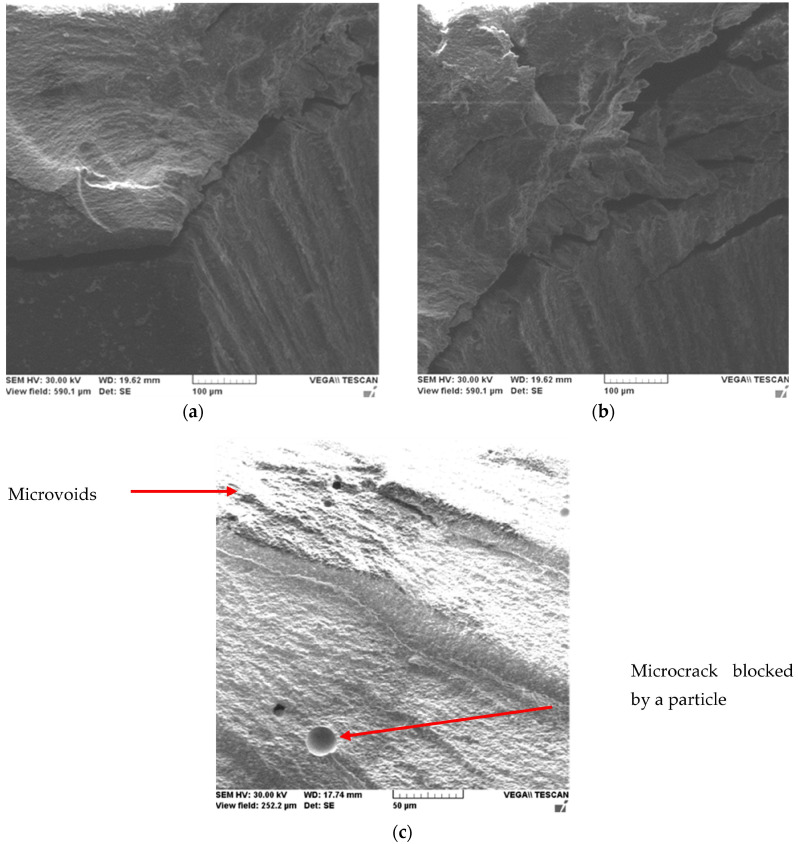
SEM microscopy of the Filtek Z250—1 sample at various magnifications 500× (**a**,**b**), 1150× (**c**) showing some micro voids resulting from the pulling out of the filler particles.

Figure 5 shows the microstructure of the Filtek Z250 composite in fracture at different scales of magnification. The characteristic of this material is the existence of microgules caused by the pulling out of the micrometer filler particles from the base matrix in which they were embedded, which could not be observed for the Filtek Supreme XT nanocomposite.

**Figure 6:**
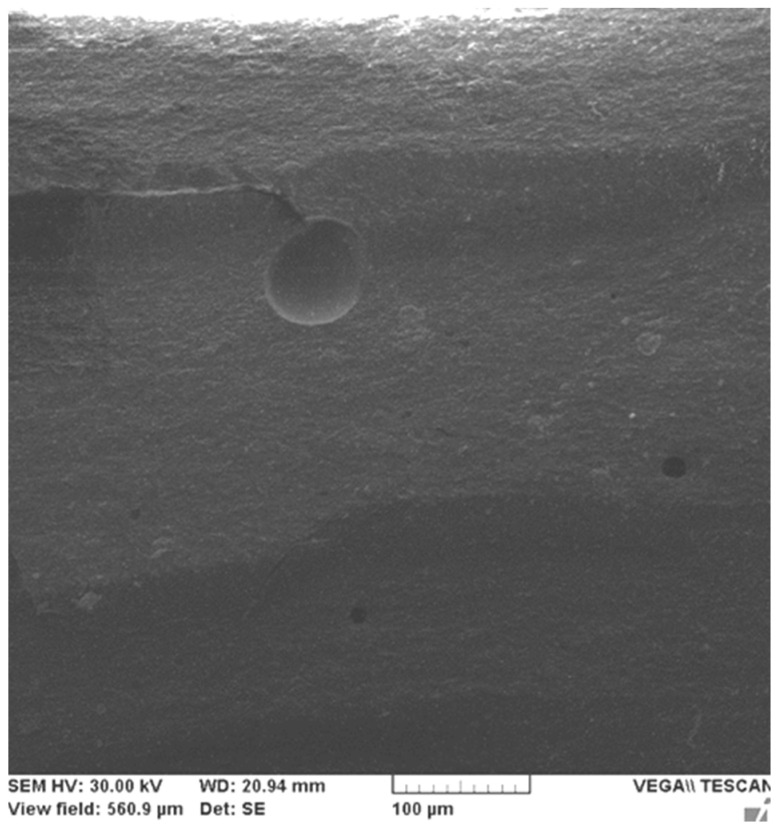
SEM microscopy of Filtek Z250-4 sample with microcrack blocked by a micrometer particle and areas with effective micro filler pull-outs.

The previously assumed mechanism of microparticle blocking of microcracks in the Filtek Z250 hybrid composite is highlighted in Figure 6. We can see a microcrack of about 150 µm, which, on reaching a relatively large particle of about 40 µm, is stopped in its advance through the material. In fact, this detail can also be seen at the bottom of Figure 5c in the marked area. We conclude that this mechanism may explain the superior properties of the Filtek Z250 hybrid composite compared to the Filtek Supreme XT nanocomposite. However, we must consider the other advantages of using nanoparticles for reinforcement: a lower viscosity in the unpolymerized state, and preservation of surface gloss over time, which are particularly important in dental restoration practice [13,14,15,16].

### 3.2. Theoretical Design Considerations

#### 3.2.1. Types of Scenarios in the Description of Dental Composite Materials Dynamics

It is a known fact that the mechanical behaviors of composite dental materials are described through material constitutive laws. For the most part, these are empirical laws that depend on the nature of the dental composite material. Moreover, the variables that describe the mechanical behaviors of said materials and play a role in these material constitutive laws can be expressed through continuous and differentiable functions. Because in time, during mastication, dental composite materials are subjected to various “efforts” (mechanical wearing, chemical wearing, etc.), they will suffer both structural and functional “transformations” (see, for example, the subsequent surface analyzes following various mechanical “efforts”). Such situations cannot be described unless using variables expressed through continuous and non-differentiable mathematical functions (fractal/multifractal functions) [6,7,8], variables that operate in the context of the SRT. Within the framework of SRT, two description scenarios are proposed to describe the mechanical behavior of dental composite materials: the Schrödinger-type scenario and the Madelung-type scenario. The two scenarios are not mutually exclusive; rather, they are complementary.

In the Schrödinger-type scenario [6,7,8], the dynamics of dental composite materials are described through the multifractal Schrödinger equation:

(1) $$\lambda^{2}{\left(dt\right)}^{\left[\frac{4}{f\left(\alpha \right)}\right]-2}\partial_{l}\partial^{l}\Psi +i\lambda {\left(dt\right)}^{\left[\frac{2}{f\left(\alpha \right)}\right]-1}\partial_{t}\Psi =0,$$

where

(2) $$\partial_{t}=\frac{\partial }{\partial_{t}},\partial_{l}=\frac{\partial }{\partial x^{l}},\partial_{l}\partial^{l}=\frac{\partial^{2}}{\partial x_{l}^{2}}.$$

In the above relations, Ψ is the states function, dt is the scale resolution, $x^{l}$ is the multifractal spatial coordinate, t is the non-multifractal temporal coordinate with the role of an affine parameter of the motion curves, λ is a parameter associated to the fractal/multifractal-non-fractal/non-multifractal scale transition, f(α) is the singularity spectrum with a singularity index of order $\alpha =\alpha (D_{F})$ and $D_{F}$ is the fractal dimension of the motion curves [17,18,19]. 

On the other hand, by choosing Ψ of the form:

(3) $$\Psi =\sqrt{\rho }e^{is}$$

where $\sqrt{\rho }$ is the amplitude and s is the phase, and introducing the real velocity fields ($V_{D}^{i}$- differentiable velocity field, $V_{F}^{i}$-non-differentiable velocity field):

(4) $$V_{D}^{i}=2\lambda (dt{)}^{\left[\frac{2}{f\left(\alpha \right)}\right]-1}\partial^{i}s$$

(5) $$V_{F}^{i}=i\lambda (dt{)}^{\left[\frac{2}{f\left(\alpha \right)}\right]-1}\partial^{i}\ln\rho$$

and the multifractal Schrödinger equation is reduced to the multifractal hydrodynamic equation system—the Madelung-type scenario:

(6) $$\partial_{t}V_{D}^{i}+V_{D}^{l}\partial_{l}V_{D}^{i}=-\partial^{i}Q$$

(7) $$\partial_{t}\rho +\partial_{l}\left(\rho V_{D}^{l}\right)=0$$

with Q the multifractal specific potential:

(8) $$Q=-2\lambda^{2}(dt{)}^{\left[\frac{4}{f\left(\alpha \right)}\right]-2}\frac{\partial^{l}\partial_{l}\sqrt{\rho }}{\sqrt{\rho }}=-V_{F}^{i}V_{F}^{i}-\frac{1}{2}\lambda (dt{)}^{\left[\frac{2}{f\left(\alpha \right)}\right]-1}\partial_{l}V_{F}^{l}.$$

Equation (6) corresponds to the multifractal specific momentum conservation law, while Equation (7) corresponds to the multifractal state density conservation law. The multifractal specific potential (8) implies the multifractal specific force:

(9) $$F^{i}=-\partial^{i}Q=-2\lambda^{2}(dt{)}^{\left[\frac{4}{f\left(\alpha \right)}\right]-2}\partial^{i}\frac{\partial^{l}\partial_{l}\sqrt{\rho }}{\sqrt{\rho }}$$

which is a measure of the multifractality of the motion curves of the dynamics.

From the Equations (6)–(8) the following meanings result:

- Any dental composite materials structural units are in permanent contact with a multifractal medium through the multifractal specific force;
- The multifractal medium can be assimilated with a multifractal fluid whose dynamics are characterized by the multifractal hydrodynamic equation system;
- The velocity field $V_{F}^{i}$ is absent from the multifractal states density conservation laws. In such a context, it induces non-manifest dental composite materials dynamics facilitating the transmission of multifractal specific momentum and multifractal energy of focus;
- In dental composite materials dynamics, the ”self-aspect” of the multifractal specific momentum, transfer the reversibility, and existence of eigenstates are guaranteed by the conservation of multifractal energy and multifractal momentum. Using the tensor:
  (10) $${\hat{\tau }}^{il}=2\lambda^{2}{\left(dt\right)}^{\left[\frac{4}{f\left(\alpha \right)}\right]-2}\rho \partial^{i}\partial^{l}\ln\rho$$

Equation (9) takes the form of a multifractal equilibrium equation:

(11) $$\rho \partial^{i}Q=\partial_{l}{\hat{\tau }}^{il}$$

Moreover, since the tensor ${\hat{\tau }}^{il}$ can also be written in the form:

(12) $${\hat{\tau }}^{il}=\eta \left(\partial_{l}V_{F}^{i}+\partial_{i}V_{F}^{l}\right)$$

with:

(13) $$\eta =\lambda {\left(dt\right)}^{\left[\frac{2}{f\left(\alpha \right)}\right]-1}\rho$$

a multifractal linear constitutive equation for a multifractal “viscous fluid”, becomes functionally offering at the same time the reason for an original interpretation of coefficient η as a multifractal dynamic viscosity of the multifractal fluid.

#### 3.2.2. Material Constitutive Laws

The previous relations, which are considered constitutive equations for deformable viscous solids of multifractal type, allow us to analyzedental composite materials behavior both in terms of compression and stretching. Then, both the multifractal tension tensor $\hat{\sigma_{il}}$, and the deformation tensor $\hat{\varepsilon_{il}}$, are characterised by the following:

(14) $$\sigma^{3}-I_{1}\sigma^{2}+I_{2}\sigma -I_{3}=0$$

for multifractal tension, and:

(15) $$\varepsilon^{3}-J_{1}\varepsilon^{2}+J_{2}\varepsilon -J_{3}=0$$

for multifractal deformation. Of these, $I_{1}$, $I_{2}$ and $I_{3}$ are multifractal invariants of $\hat{\sigma_{il}}$, and $J_{1}$, $J_{2}$ along with $J_{3}$ are multifractal invariants of $\hat{\varepsilon_{il}}$. Now let us define their functional dependency with the relation:

(16) $$\hat{\sigma }=\hat{\sigma }\left(\hat{\varepsilon }\right)$$

which implies correlations between the invariants mentioned above. These correlations can be explained through the homographic transformation [20,21]:

(17) $$\varepsilon_{k}=\frac{\alpha \sigma_{k}+\beta }{\gamma \sigma_{k}+\delta },k=1,2,3$$

where $\sigma_{k}$ and $\varepsilon_{k}$ are the roots of the previously mentioned equations. The coefficients α, β, γ, and δ gain the status of material parameters, while the matrix:

(18) $$\hat{M}=\left(\begin{array}{cc} \alpha & \beta \\ \gamma & \delta \end{array}\right)$$

given by Equation (17) becomes fundamental in generating the material constitutive laws through the differential geometry associated with this matrix.

In such a context, we will obtain a relation between the matrix and an ensemble of values of σ for which $\sigma^{\prime }$ remains constant. From a geometric perspective, this means to find the ensemble $\left(\alpha ,\beta ,\gamma ,\delta \right)$ which corresponds unequivocally to σ. Using Equation (16) the problem proves to be reducible to solving the Riccati-type differential equation [20,21]:

(19) $$d\sigma +\omega_{1}\sigma^{2}+\omega_{2}\sigma -\omega_{3}=0$$

where the following notations were used:

(20) $$\omega_{1}=\frac{\gamma d\alpha -\alpha d\gamma }{∆},\omega_{2}=\frac{\delta d\alpha -\alpha d\delta +\gamma d\beta -\beta d\gamma }{∆},\omega_{3}=\frac{\delta d\beta -\beta d\gamma }{∆}$$

(21) ∆=αδ−βγ.

It is possible to see that the metric:

(22) $${ds^{2}=\left(\frac{\delta d\alpha -\alpha d\delta +\gamma d\beta -\beta d\gamma }{∆}\right)}^{2}-\frac{d\alpha d\delta -d\beta d\gamma }{∆}$$

is in direct relation to the discriminant of Equation (18):

(23) $$ds^{2}=\frac{1}{4}\left({\omega_{2}}^{2}-4\omega_{1}\omega_{3}\right).$$

The three differential 1-forms of Equation (19) constitute a coframe in any point of absolute space. This allows the translation of geometric properties of the absolute space into algebraic properties of Equation (18). The simplest of these refers to dynamics over the geodesics of the metrics of Equation (22), which can be translated directly to statistical properties, given that we discuss multifractalization through stochasticity. In this situation, the 1-forms $\omega_{1}$, $\omega_{2},$ and $\omega_{3}$ are exact differentials of the same parameter, which is the length of the arc of the geodesic. Let us note it with s. Explicitly, we obtain:

(24) $$\omega_{1}=a_{1}ds,\omega_{2}=a_{2}ds,\omega_{3}=a_{3}ds$$

where $a_{1}$, $a_{2}$ and $a_{3}$ are constants which characterize a certain geodesic of the given family. Along this geodesic, Equation (18) becomes a differential equation of the type:

(25) $$\frac{d\sigma }{ds}=a_{1}\sigma^{2}+2a_{2}\sigma +a_{3}$$

Equation (24) admits a direct integration, giving the following solutions:

(26a) $$\sigma \left(s\right)=-\frac{\alpha_{2}}{\alpha_{1}}+\frac{\sqrt{∆}}{\alpha_{1}}\tan\left[\sqrt{∆}\left(s-s_{0}\right)\right]\;for\;∆>0$$

(26b) $$\sigma \left(s\right)=\frac{as+b}{cs+d}\;for\;∆=0$$

(26c) $$\sigma \left(s\right)=-\frac{\alpha_{2}}{\alpha_{1}}+\frac{\sqrt{∆}}{\alpha_{1}}\coth\left[\sqrt{∆}\left(s-s_{0}\right)\right]\;for\;∆<0$$

with:

(27) $$∆=a_{1}a_{3}-{a_{2}}^{2},a_{2}=\sqrt{∆}\tan\left(\sqrt{∆}s_{0}\right)$$

Furthermore, $s_{0}$, a, b, c, d are constants, not all of them are arbitrary.

Any of Equation (26) describes a deformation process for constant tensions. A similar procedure can be applied to $\hat{\varepsilon_{il}}$. Then, the following equation is satisfied:

(28) $$\frac{d\varepsilon }{d\tau }=\overset{–}{a_{1}}\varepsilon^{2}+2\overset{–}{a_{2}}\varepsilon +\overset{–}{a_{3}}$$

these constants characterize a certain geodesic of the family and τ the length of the arc. Equation (27) admits direct integration, which yields three possibilities:

(29a) $$\varepsilon \left(\tau \right)=-\frac{\overset{–}{\alpha_{2}}}{\overset{–}{\alpha_{1}}}+\frac{\sqrt{\overset{–}{∆}}}{\overset{–}{\alpha_{1}}}\tan\left[\sqrt{\overset{–}{∆}}\left(\tau -\tau_{0}\right)\right]\;for\;\overset{–}{∆}>0$$

(29b) $$\varepsilon \left(\tau \right)=\frac{\overset{–}{a}\tau +\overset{–}{b}}{\overset{–}{c}\tau +\overset{–}{d}}\;for\;\overset{–}{∆}=0$$

(29c) $$\varepsilon \left(\tau \right)=-\frac{\overset{–}{\alpha_{2}}}{\overset{–}{\alpha_{1}}}+\frac{\sqrt{\overset{–}{∆}}}{\overset{–}{\alpha_{1}}}\coth\left[\sqrt{\overset{–}{∆}}\left(\tau -\tau_{0}\right)\right]\;for\;\overset{–}{∆}<0.$$

Here, $\overset{–}{∆}$ and $\tau_{0}$ are given through the relations:

(30) $$\overset{–}{∆}=\overset{–}{a_{1}}\overset{–}{a_{3}}-{\overset{–}{a_{2}}}^{2},\overset{–}{a_{2}}=\sqrt{\overset{–}{∆}}\tan\left(\sqrt{\overset{–}{∆}}\tau_{0}\right).$$

However, these constants are not necessarily arbitrary. Now, if both σ(s) and τ(ε) follow the same manifold, which implies:

(31) $$a_{1}=\overset{–}{a_{1}},a_{2}=\overset{–}{a_{2}},a_{3}=\overset{–}{a_{3}},\tau =\frac{s}{E},E=const.$$

then from Equations (24) and (27) the following differential relation is obtained:

(32) dσ=Edε.

From Equation (31), the Young-type differential elasticity modulus results:

(33) $$E=\frac{d\sigma }{d\varepsilon }.$$

This equation can also present negative values, $E=\frac{d\sigma }{d\varepsilon }<0$, which might specify self-structuring phenomena through pattern formation. Now, if we admit the particular case:

(34) $$a_{1}=-f,2a_{2}=f,a_{3}=0.$$

Equation (24) is reduced to a logistic-type equation [17,18]:

(35) $$\frac{d\sigma }{ds}=f\sigma \left(1-\sigma \right)$$

thus, the growth of σ is limited by the finite matrix effect. The solution of Equation (34) is:

(36) $$\sigma =\frac{1}{1-\left(1-\frac{1}{\sigma_{0}}\right)e^{\left(-fs\right)}}$$

where $\sigma_{0}$ is an integration constant. A similar mathematical procedure can be applied to Equation (27), which implies:

(37) $$\varepsilon =\frac{1}{1-\left(1-\frac{1}{\varepsilon_{0}}\right)e^{\left(-\Omega \tau \right)}}$$

where $\varepsilon_{0}$ is an integration constant. Such a result is possible if, in Equation (27), we admit the identifications:

(38) $$\overset{–}{a_{1}}=-\Omega ,2\overset{–}{a_{2}}=\Omega ,\overset{–}{a_{3}}=0.$$

**Figure 7:**
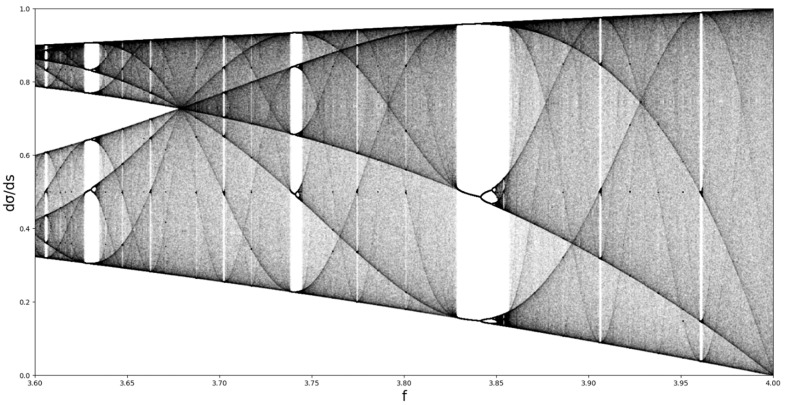
Representation of Equation (35) in the same manner as the logistic map; chaotic region with areas of stability.

It is possible to highlight the otherwise well-known fact of nonlinear dynamics [17,18] that the increase in the logistic map parameter after 3.56995 leads to chaos. This means that between roughly 3.6 and 4, there are complex chaotic dynamics; in our case, it means that the variation of f between 3.6 and 4 leads to repeated ordered-chaotic dynamics transition for $\frac{d\sigma }{ds}$. Practically, because of the nature of this mapping, there are order-disorder transitions in dental composite materials dynamics, wherein f is the control parameter (see Figure 7).

In order to further investigate the dental composite materials dynamics of this system, it is also possible to rewrite Equation (19) as:

(39) $$\dot{w}-\frac{1}{M}w^{2}+2\frac{R}{M}w-K=0.$$

It is important to find the most general solution of this equation. For our current needs, it is enough to note that the complex number roots of the quadratic polynomial of Equation (39):

(40) $$w_{0}\equiv R+iM\Omega ,w_{0}^{*}\equiv R-iM\Omega ;\Omega^{2}=\frac{K}{M}-{\left(\frac{R}{M}\right)}^{2}$$

are constant solutions of the equation, thus their derivative is zero. Let us perform the homographic transformation:

(41) $$z=\frac{w-w_{0}}{w-w_{0}^{*}}$$

and now it can easily be seen by direct calculation that z is a solution of the linear and homogeneous equation of the first order:

(42) $$\dot{z}=2i\Omega z\;∴\;z(t)=z(0)e^{2i\Omega t}.$$

Therefore, if we conveniently express the initial condition z(0), we can give the general solution of the Equation (39) by simply inverting the transformation in Equation (41), with the result:

(43) $$w=\frac{w_{0}+re^{2i\Omega \left(t-t_{r}\right)}w_{0}^{*}}{1+re^{2i\Omega \left(t-t_{r}\right)}}$$

where *r* and $t_{r}$ are two real constants that characterize the solution. Using Equation (40), we can put this solution in real terms, which highlights a frequency modulation through what we would call a Stoler transformation [20,21] which leads us to a complex form of this parameter. Furthermore, if we make the notation:

(44) r≡coth⁡τ

the real term becomes:

(45) z=R+MΩh

where h is given by:

(46) $$h=-i\frac{cosh\tau -e^{-2i\Omega \left(t-t_{m}\right)}sinh\tau }{cosh\tau +e^{-2i\Omega \left(t-t_{m}\right)}sinh\tau }.$$

The meaning of this complex parameter will become clear later. For the moment, let it be noted that any dynamic process appears here as a frequency modulation process using a gauge invariance of a Riccati-type.

In these figures, Real (h) (the amplitude at various scale resolutions given by the maximum value of Ω) is represented as functions of t and Ω for r = 0.5.

**Figure 8:**
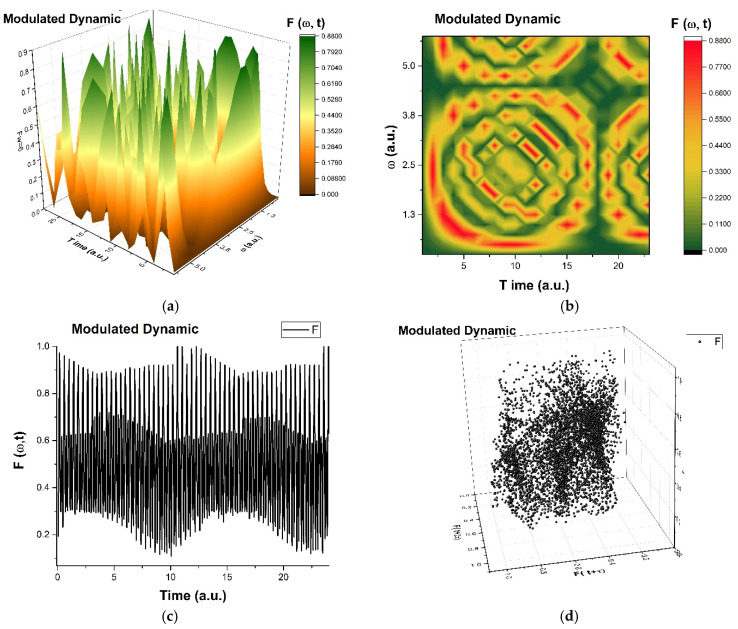
(**a**–**d**): The ”modulated dynamic modes” in mastication of the structural units of dental composite materials dynamics are presented: (**a**)—3D diagram, (**b**)—contour diagram, (**c**)—time series and (**d**)—reconstituted attractor for scale resolutions given by Ω_max_.

**Figure 9:**
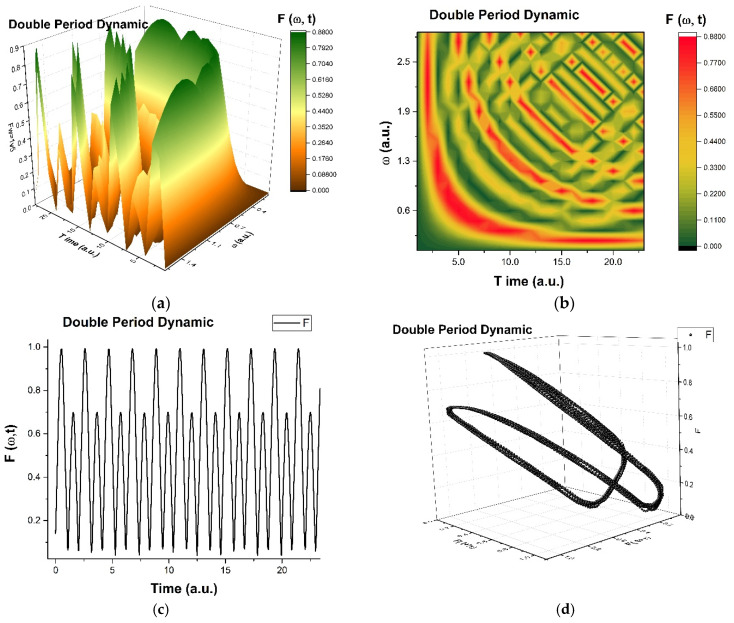
(**a**–**d**): The ”double period dynamic modes” in mastication of the structural units of dental composite materials dynamics are presented: (**a**)—3D diagram, (**b**)—contour diagram, (**c**)—time series and (**d**)—reconstituted attractor for scale resolutions given by Ω_max_.

As is observed in Figure 8a–d and Figure 9a-d, the natural transition of dental composite materials dynamics passes through various states, such as self-modulation and period doubling. The dental composite materials dynamics never reach a chaotic state, but they permanently evolve towards that state. 

**Figure 10:**
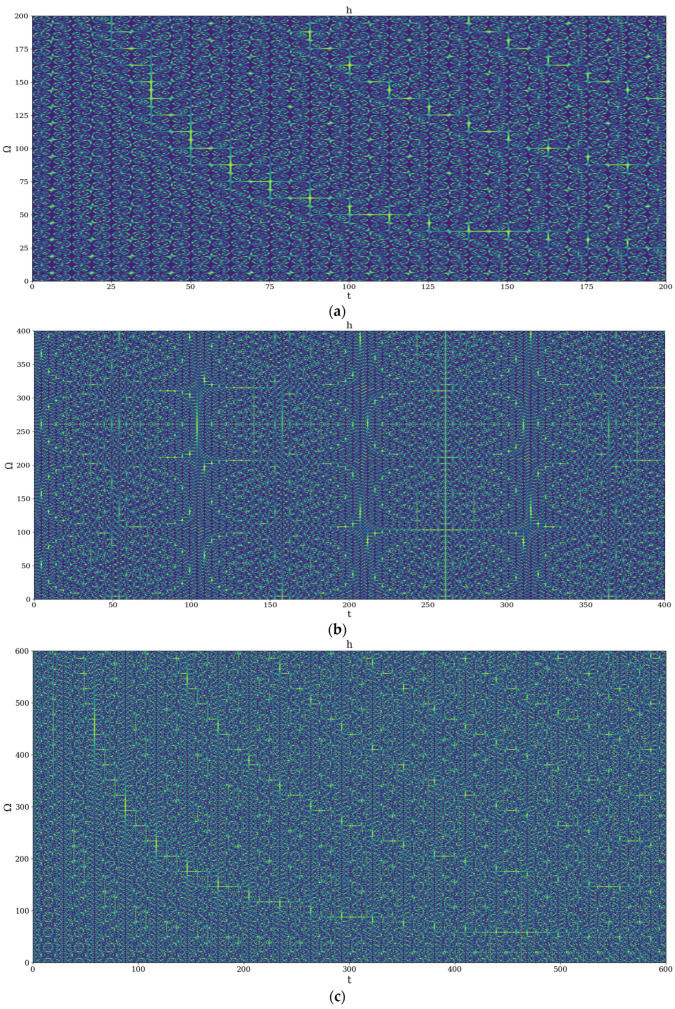
(**a**): Example of 2D plot fracture patterns by means of $h\left(\Omega ,t\right)$, maximum at 200; Φ=2.35. (**b**): Example of 2D plot fracture patterns by means of $h\left(\Omega ,t\right)$, maximum at 400; Φ=2.35. (**c**): Example of 2D plot fracture patterns by means of $h\left(\Omega ,t\right)$, maximum at 600; Φ=2.35.

Let it be noted that the mathematical formalism of the SRT naturally implies various operational procedures (invariance groups, harmonic mappings, groups isomorphism, embedding manifolds, etc.) with several applications incomposite materials dynamics [7,8]. Interestingly, plotting h in dimensionless parameters again highlights certain temporal self-similar properties, with the multifractal structures being contained into similar multifractal structures at much higher scales (Figure 10a–c). This behavior is quite difficult to represent because of the complicated balance between choosing an adequate number of plot points and manifesting self-similarity. Still, it shows how the small-scale behavior of the system ripples and manifests itself at higher scales, which is exactly what we would expect from a multifractal system. 

In such a context, the transitions from the patterns presented in Figure 8b and Figure 9b through Figure 10a–c can be made through compression. Figure 9a–c illustrates fracture patterns due to compressive stresses. Moreover, the phenomenon can be explained through a mathematical equation that governs it.

Thus, an analytical expression is given for the nonlinear range of σ−ε curves under compression and is as follows (see Equation (33), through a convenient choice of the parameters and considering that the dental composite materials dynamics manifest on the same manifold, which implies s≡τ):

(47) $$\sigma =E_{0}\varepsilon \left(1+\alpha \varepsilon \right)$$

where $E_{0}$ is the initial tangent at zero (corresponding to the elastic modulus for purely linear elastic behavior). For example, for fused silica, the elastic material parameters are commonly known and need no further elaboration. The used (linear) elastic modulus is $E_{0}$ = 70 GPa. Elastic volume compressibility is expressed by the Poisson ratio with ν = 0.17. In the equation, the experimental data provided the coefficient α = 3 for the nonlinear behavior. Such a law, in dimensionless coordinates (for various fractal dimensions and scale resolutions), can very well describe the experimental curves obtained in Figure 1 and Figure 2. Let it be noted that such scenarios were employed before in the description of mechanical behaviors of various materials. For example, in [22], a Schrödinger type scenario was used for the description of the hysteresis-type behavior of shape memory alloys.

## 4. Materials and Methods

### Experimental Design

The equipment for tensile/compression testing of dental composite materials is the INSTRON 3382 USA servo-hydraulic type. It is characterized by the following technical parameters:

○ load capacity of 100 kN with a maximum speed of 500 mm/min, minimum speed of 5 × 10^−3^ mm/min;
○ maximum force at maximum speed: 50 kN;
○ maximum speed at maximum force: 250 mm/min;
○ return speed: 600 mm/min;
○ Blue hill^®^ Lite software;
○ INSTRON climatic enclosure with a possible temperature range of −70 °C to +350 °C;
○ INSTRON 3-point bending device, 100kN, and a compression device.

The equipment, connected and controlled by a computer, can develop different compression speeds of materials, in this case, dental composites to follow their behavior in different compression phases.

The composite resin tested in the present study and their structure are presented in Table 2. The samples from the two materials were made into cylindrical molds with a length of 9 mm and a diameter of 4.5 mm, i.e., with a length/diameter (l/d) ratio of 2:1, which is standard for this type of mechanical test. They were subjected to compression tests at different working speeds of the mechanical equipment, of 0.5, 1, and 1.5 mm/min. The tests were carried out at a room temperature of 27 °C.

After the mechanical tests were completed, the resulting fragments from the samples were investigated by SEM microscopy in order to identify the causes of breakage and the mode of propagation of the microcracks.

Samples made from the two materials were machined into cylindrical form with a length of 9 mm and a diameter of 4.5 mm, i.e., with a length/diameter (l/d) ratio of 2:1, which is standard for this type of mechanical test. They were subjected to compression tests at different working speeds of the mechanical equipment of 0.5, 1, and 1.5 mm/min. The tests were carried out at room temperature, 27 °C.

After the mechanical tests, the samples were investigated by SEM microscopy to identify the causes of breakage and the mode of propagation of microcracks. Such experimental studies have been performed on other materials and employed other types of mechanical “efforts” (see, for example, [23,24]). 

## 5. Conclusions

The main conclusions of the present paper are the following:

Regarding the experimental design, the compressive strength of the Filtek Supreme XT composite was obtained as 332.14 MPa, lower than that of the Filtek Z250 material, 392 MPa, values in agreement with those specified by the manufacturer and the literature. However, this parameter depends on the samples’ compaction before light curing. Analysisof the compressive stress—compressive strain dependencies revealed stronger cracking of the Filtek Supreme XT composite than Filtek Z250 prior to fracture, which has important clinical implications for ensuring the integrity of dental restorations. SEM microscopy results of the two materials showed microcracks produced upon compression and, in the case of Filtek Z250, the existence of micro gaps produced by the detachment of micrometer filler particles. A crack-blocking mechanism by the filler microparticles of Filtek Z250 has been revealed. This may explain its superior mechanical properties compared to the Filtek Supreme XT nanocomposite, where cracks bypass the nanometer particles.

Regarding the theoretical design, in Schrödinger-type and Madelung-type scenarios, the descriptions of dental composite materials dynamics are highlighted. The existence of an SL(2R) type symmetry allows the generation of material constitutive laws. In such a context, through gauge invariances of Riccati-type, various non-linear behaviors are established: double period and modulated dynamics regimes. A material constitutive law of Hooke-type in differential form was obtained, which was explained for compression-type behaviors. In such a context, an explicit form of the law was highlighted, which was correlated with the experimental data.

## Figures and Tables

**Table 1:** Experimental values of shear strain and compressive strength for Filtek Supreme XT and Filtek Z250 composite specimens.

No. crt.	Sample	Ultimate Compressive Strain (%)	Ultimate Compressive Strength (MPa)
1	Filtek Supreme XT-1	10.45	82
2	Filtek Supreme XT-2	11.18	97.41
3	Filtek Supreme XT-3	15.79	333.12
4	Filtek Supreme XT-4	13.61	205.19
5	Filtek Supreme XT-5	12.66	177.18
6	Filtek Supreme XT-6	14.35	172.83
7	Filtek Supreme XT-7	10.55	213.81
8	Filtek Z250-1	16.45	194.58
9	Filtek Z250-2	15.54	392
10	Filtek Z250-3	16.63	314.72
11	Filtek Z250-4	11.84	164.39
12	Filtek Z250-5	9.62	53.08

**Table 2:** Structure of the two materials.

Material	Manufacturer	Type/Shade	Matrix	Filler
Filtek Supreme XT Universal Restorative	3MESPE, St. Paul, MN, USA	Nanocomposite/A2	Bis-GMA, Bis-EMA, TEGDMA UDMA	Non-agglomerated/non-aggregated 20 nm silica particles, Non-agglomerated/non-aggregated 4 to 11 nm zirconia particles, Aggregated zirconia/silica cluster filler (comprised of 20 nm silica and 4 to 11 nm zirconia particles) 78.5 wt % 63.3 vol%
Filtek Z250 Universal Restorative	3MESPE, St. Paul, MN, USA	Microhybrid/A2	Bis-GMA, Bis-EMA, TEGDMA UDMA	Silica and zirconia particles 0.01–3.5 µm, average size 0.6 µm 84.5 wt% 60 vol%

## Data Availability

Research data is available upon request.

## Abbreviations

List of abbreviations:

SRT	Scale Relativity Theory
Bis-GMA	bisphenol A-glycidyl methacrylate
bis-EMA	bisphenol A-ethoxylateddimethacrylate
TEGDMA	triethyleneglycoldimethacrylate
UDMA	urethane dimethacrylate,
